# Allostery through protein-induced DNA bubbles

**DOI:** 10.1038/srep09037

**Published:** 2015-03-12

**Authors:** Joseph J. Traverso, Valipuram S. Manoranjan, A. R. Bishop, Kim Ø. Rasmussen, Nikolaos K. Voulgarakis

**Affiliations:** 1 Department of Mathematics, Washington State University, Richland, WA 99354, USA; 2 Department of Mechanical Engineering, Washington State University, Richland, WA 99354, USA; 3 Department of Mathematics, Washington State University, Pullman, WA 99163, USA; 4 Theoretical Division, Los Alamos National Laboratory, Los Alamos, NM 87545, USA

## Abstract

Allostery through DNA is increasingly recognized as an important modulator of DNA functions. Here, we show that the coalescence of protein-induced DNA bubbles can mediate allosteric interactions that drive protein aggregation. We propose that such allostery may regulate DNA's flexibility and the assembly of the transcription machinery. Mitochondrial transcription factor A (TFAM), a dual-function protein involved in mitochondrial DNA (mtDNA) packaging and transcription initiation, is an ideal candidate to test such a hypothesis owing to its ability to locally unwind the double helix. Numerical simulations demonstrate that the coalescence of TFAM-induced bubbles can explain experimentally observed TFAM oligomerization. The resulting melted DNA segment, approximately 10 base pairs long, around the joints of the oligomers act as flexible hinges, which explains the efficiency of TFAM in compacting DNA. Since mitochondrial polymerase (mitoRNAP) is involved in melting the transcription bubble, TFAM may use the same allosteric interaction to both recruit mitoRNAP and initiate transcription.

Precise communication between DNA-binding proteins is critical for many life processes, including the transcription, replication, and organization of DNA. In all of these cases, appropriate proteins form clusters, required to either initiate or execute the entire process. Although the origin of such protein assemblies is unclear, they are often assumed to be driven by direct protein–protein interactions. This assumption limits the role of DNA to simply facilitating the presence of proteins through protein–DNA interactions. Very recently, however, it has been shown that DNA may play a more active role in its own functions^1^. It has been demonstrated both experimentally^1^,^2^ and computationally^3^,^4^ that DNA deformations induced by binding proteins affect the affinity of other nearby proteins. In other words, allosteric signaling through DNA is also possible^5^. Most related studies have been restricted to two types of conformational DNA changes: stretching and bending^1^,^2^,^4^,^6^,^7^. In this work, we examine how local protein-induced unwinding of the double strand (bubbles) can also facilitate a different type of allosteric signaling. Since the local melting of DNA increases its flexibility^8^ and also exposes the genetic code to RNA polymerase, such an allosteric signal potentially regulates both transcription and gene compaction.

Mitochondrial transcription factor A (TFAM) is an excellent example to test such a hypothesis since there is strong evidence that it locally unwinds mtDNA^9^,^10^,^11^. Structurally, TFAM consists of two high mobility group (HMG) box domains A and B connected with a linker and ending with a C-terminal tail attached to Box B^12^ (Fig. 1a). TFAM binds specifically close to the light strand promoter (LSP) and heavy strand promoter (HSP1) to form the transcriptional machinery by recruiting transcription factor B2 (TFB2M) and mitochondrial polymerase (mitoRNAP)^9^,^13^,^14^,^15^,^16^. TFAM also binds nonspecifically and plays a critical role in mtDNA compaction^17^,^18^. The physical mechanism behind the dual function of TFAM is still unclear. Very recent experimental studies have shown that both specific and non-specific binding introduce a sharp U-turn in the mtDNA^18^,^19^,^20^,^21^,^22^,^23^, which, although seemingly vital in forming and appropriately orienting the transcription machinery, does not explain the high efficiency of DNA compaction in the presence of TFAM^18^. Instead, the ability of TFAM to slide rapidly on mtDNA and, upon colliding, to form stable and immobile oligomers seems to be directly related to mtDNA compaction^17^. It has been proposed that such an aggregation of two TFAM proteins melts a region of two to three base pairs (bp) at the point of contact, thus creating fixed flexible hinges that enhance mtDNA flexibility^17^. However, very recent high-resolution experiments have revealed that TFAM oligomers are neither stable nor immobile^24^. It is thus unclear how such highly diffusive hinges of limited lifetime could effectively compact mtDNA molecules.

The ability of TFAM to unwind mtDNA at the end of each HMG box (see Fig. 1b) has two critical consequences. First, it creates two flexible hinges that can potentially increase the flexibility of the DNA. Second, it effectively generates an attractive interaction that drives TFAM oligomerization. We show that the mechanism underlying this allosteric interaction can be an unbalanced force created by the coalescence of two TFAM-induced bubbles. The role of *thermally* induced local openings of the double strand appears to be critical, since it affects both the transmission of the allosteric signal and the stability of the aggregations. The main result of TFAM oligomerization is excitation of a considerably larger bubble (hinge) at the point of contact of two TFAMs, which increases mtDNA flexibility even further and regulates compaction. Interestingly, TFAM binds specifically about 20 bp away from the transcription starting point, which, as we show below, is within the range of the allosteric attraction of two bubbles. Since both TFB2M and mitoRNAP are involved in exciting the transcription bubble^25^, TFAM can help the two proteins excite the transcription bubble and then a coalescence of the transcription bubble and a TFAM bubble could stabilize the transcription machinery.

## Results

To avoid computationally expensive atomistic molecular dynamics simulations, we use the extended Peyrard–Bishop–Dauxois (EPBD) model to describe the local melting dynamics of DNA. EPBD is a one-dimensional (1D) mathematical model with a demonstrated capability for reproducing experimental results on both the mechanical and thermal denaturation of DNA^26^,^27^,^28^,^29^,^30^,^31^. The potential energy of the EPBD model is:
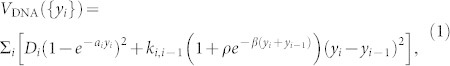
where *y_i_* describes the distortion of the *i*th base pair from its equilibrium position. The hydrogen bonds of a base pair are modeled by Morse potentials (first term in Eq. (1)), while the stacking interactions are described by nonlinear springs (second term in Eq. (1)). The model, although simple, takes into account the sequence specificity that is reflected in the parameters *D_i_*, *a_i_*, *k_i_*_,*i* − 1_, *ρ*, and *β*. In this study, we will use the values of the parameters in Ref. 27, which have been adjusted to reproduce a variety of experimental observations.

**Figure 1:**
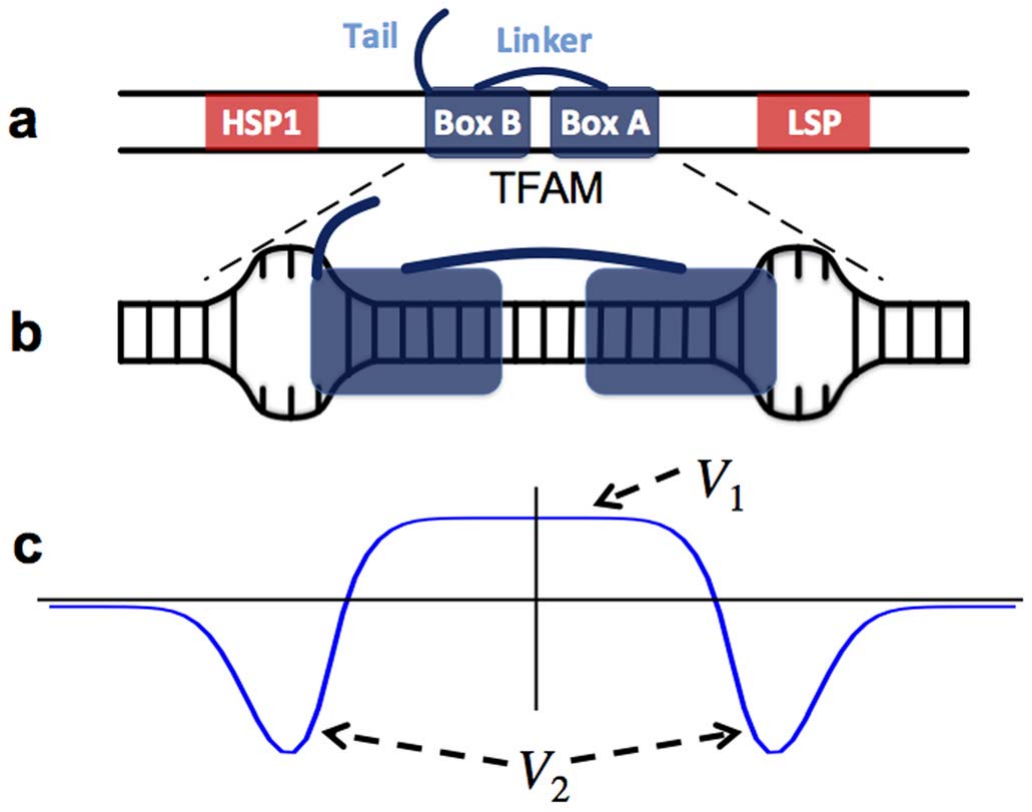
TFAM-DNA interaction model. (a) Schematic representation of the TFAM structure. Note the orientation of TFAM on DNA relatively to HSP1 and LSP promoters. (b) Illustration of TFAM-DNA complex and the resulting local unwinding of the double helix at the end of each HMG Box domain. (c) Representation of the DNA-TFAM interaction potential (see Eq. 2). *V*_1_ suppresses the double strand at the core of the TFAM-DNA complex and *V*_2_ unwinds it at the two ends of the TFAM.

The sliding of TFAMs on DNA is assumed to be purely 1D. The interaction between the protein and DNA has two parts:
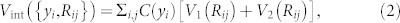
where *R_ij_* = *r_j_* − *ia* is the distance of the center of the *j*th protein from the *i*th base pair and *a* is the distance between two consecutive base pairs. The first part of the equation, *V*_1_(*R_ij_*) = *A*_1_{tanh[*γ*_1_(*σ*/2 − *R_ij_*)] + tanh[*γ*_1_(*σ*/2 + *R_ij_*)]}, describes the interaction of the binding protein with the DNA backbone, which slightly suppresses the base pair^32^. Here, *σ* denotes the size of the protein. The second part, *V*_2_ = −*A*_2_{exp[−*γ*_2_(*σ*/2 − *r*)^2^] + exp[−*γ*_2_(*σ*/2 + *r*)^2^]}, models the ability of the TFAM to unwind the DNA at the end of the two HMG box domains^9^,^10^,^11^. The coefficient *C* = tanh[*γy_i_*], where *γ* = 1 Å^−1^, controls the strength of the interaction. It increases linearly until the base pair opens (*y_i_* ≥ 2 Å) and then it plateaus. A schematic representation of the interaction potential is presented in figure 1c.

Since experiments indicate that TFAM proteins do not form oligomers in the absence of DNA (see Ref. 18, for instance), we neglect any possible direct attraction and use a Weeks–Chandler–Andersen (WCA) potential^33^ to describe the repulsion (soft sphere) between two TFAMs:
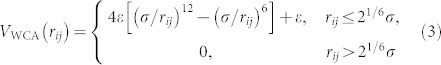
where *r_ij_* is the distance between the centers of the *i*th and *j*th proteins, and *ε* is the interaction strength. The total direct protein-protein interaction energy of a system of multiple proteins is
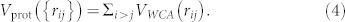


As explained below, the parameters *A*_1_, *A*_2_, *γ*_1_, *γ*_2_, and *ε* of equations (2) and (3) have been adjusted to reproduce the experimentally observed cooperative binding of TFAM^17^ (see Methods). The protein size is assumed to be *σ* = 28 bp, an estimate that is in good agreement with most experimental observations^17^,^18^. To study the behavior of this TFAM–DNA model, we perform Langevin dynamics simulations at a temperature T = 300 K (see Methods). The potential energy of equation (2) melts a segment three to four bp long at the end of each HMG box.

### Bubble-mediated allosteric protein–protein interaction

**Figure 2:**
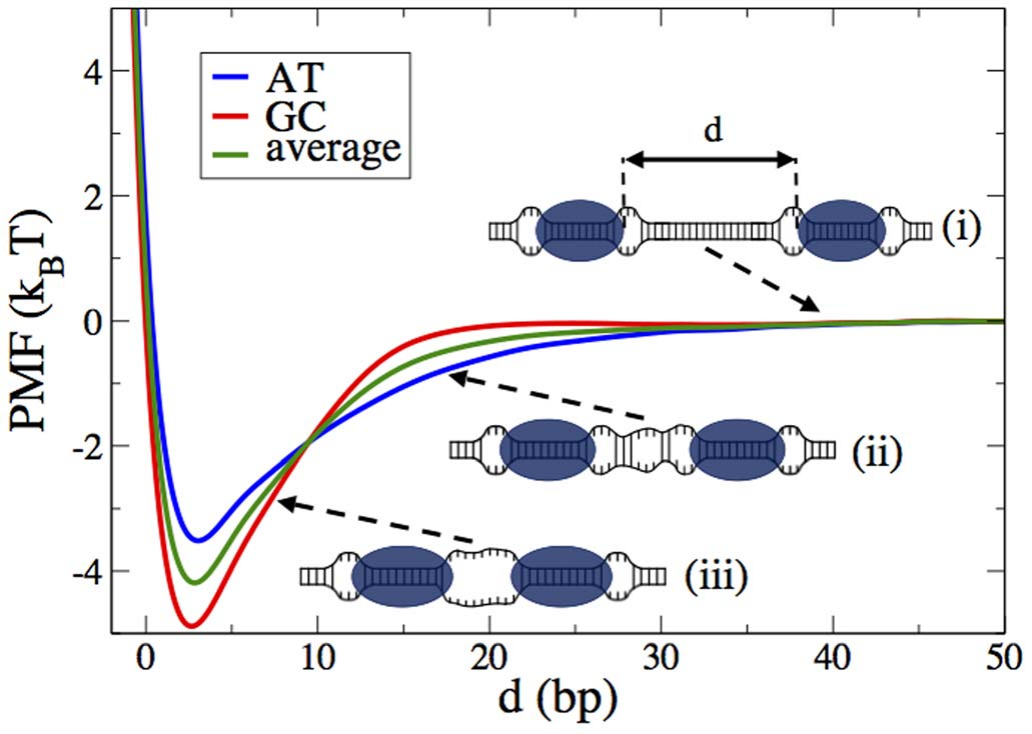
Allosteric protein-protein interaction. PMF of a TFAM dimer as a function of the surface-to-surface distance *d* for a homogeneous AT (blue) and GC (red) DNA molecule. The green line corresponds to the average of the two profiles. The minimum of all three profiles is located at *d*_0_ = 3 bp. Insets schematically show the role of thermally induced base pair openings in dimer formation. (i) For *d* > 40 bp the two proteins practically do not feel the presence of each other. (ii) For *d* < 20 bp thermally induced spontaneous base pair openings create a tunnel of partially open base pairs that connects the two proteins. This produces an unbalance force that drives proteins collapse. (iii) For *d* < 10 bp the area between the two proteins is completely melted thereby creating a flexible hinge that increases DNA's flexibility.

To test our hypothesis that the coalescence of bubbles drives protein aggregation, we perform standard potential of mean force (PMF) calculations (Methods). Figure 2 presents the PMF between two TFAMs in a homogeneous AT and GC molecule, which, as predicted, has an attractive structure. Protein aggregation is triggered by spontaneous thermal openings in the double strand. These openings (or thermal bubbles) exist even at temperatures well below the melting transition and are a result of the interplay between entropy, nonlinearity, and sequence specificity^26^,^28^,^34^. The communication between two proteins begins when they diffuse to positions where a spontaneous bubble nucleation of length approximately equal to their surface-to-surface distance is possible (Fig. 2 ii). This local thermal melting reduces the system's total free energy and creates an unbalanced force that pushes the two proteins toward each other (Fig. 2 iii). This represents a new type of allostery initiated by protein-induced bubbles and transmitted through thermal bubbles.

The depth (~4.23 k_B_T), the average surface-to-surface distance (~10 bp), and the range (~20 bp) are three of the main characteristics of the allosteric potential presented in Figure 2. The parameters of equations (2) and (3) were tuned so that the depth provides a cooperative factor of ~70, as estimated in the experimental work of Ref. 17. The coalescence of two small TFAM-induced DNA bubbles can be viewed as the elimination of two half-bubbles, i.e. two forks, from the system. The activation energy of such small forks is associated with the energy cost to unzip a base pair. Thus, the elimination of two forks lowers the free energy of the system by approximately the depth of the interaction potential presented in Figure 2. The average surface-to-surface area includes approximately 10 melted bps, which indicates that TFAM oligomerization provides an additional and significantly larger flexible hinge than the flexible hinge of a monomer would. Thus, TFAM oligomerization could potentially increase the flexibility of a DNA molecule and consequently regulate DNA compaction^17^,^24^,^35^,^36^. In the limit of maximum coverage of DNA by TFAM the energetically most favorable hinge is 3 bp, i.e. equal to the surface-to-surface distance, *d*_0_, that corresponds to the minimum of PMF (see Fig. 2). Interestingly, this 3 bp melted segment in the limit of high TFAM concentration was also predicted by the authors of Ref. 17 using an independent calculation based on the counter length of DNA. Based on our analysis, the effective size of TFAM is *σ*_eff_ = *σ* + *d*_0_ or *σ*_eff_ = 31 bp and the maximum number of TFAMs a DNA molecule can host is *L*_DNA_/*σ*_eff_, where *L*_DNA_ is the length of the DNA. According to Figure 2, a TFAM can attract another TFAM or other proteins from a distance of approximately 20 bp. This result is particularly important when we discuss below the role of TFAM in transcription initiation. The PMF is also sequence dependent. We see that, in homogeneous AT DNA molecules, the range of the potential is longer than in homogeneous GC molecules; however, GC regions support more stable dimerization. Thus, in a realistic DNA molecule, AT-rich regions can facilitate the long-distance transmission of allosteric signals, while GC regions provide a more stable aggregation.

### Reversibly assembled protein aggregates

Due to the finite depth of the interaction potential, the picture of multiple TFAMs sliding on mtDNA is expected to be a typical example of 1D reversible particle–particle aggregation. In such systems, one expects oligomerization and dissociation events, as well as a reduction of mobility due to oligomerization, crowding, or even dynamically arrested states^37^. In general, large bubbles induced by TFAM oligomerization are expected to contribute more to DNA compaction than small hinges. However, their excitation, lifetime, and mobility ultimately determine their effectiveness. A large bubble with a short lifetime or high diffusivity, for instance, would have a very small probability of fully developing and melting DNA at a certain position. The question is, however, to what extent does our model agree with recent experimental observations and, in particular, the data presented in Refs. 17, 18, 24?

**Figure 3:**
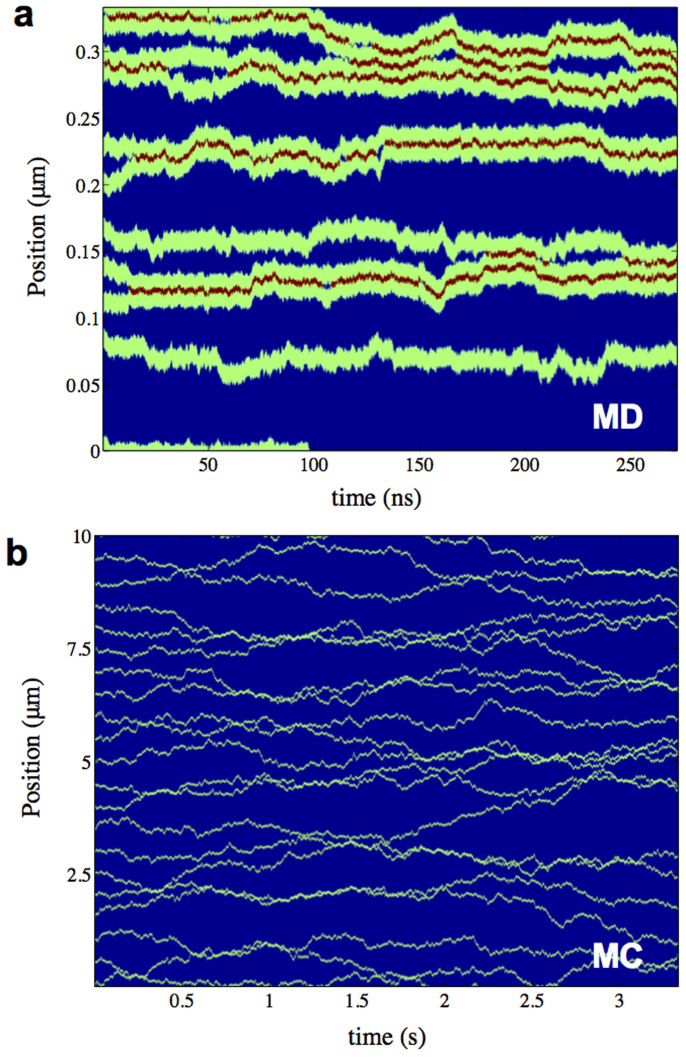
TFAM diffusion and oligomerization on DNA. (a) Langevin dynamics of 10 TFAMs diffusing in a 0.33 μm long mtDNA. Green color shows the envelope and position of TFAMs as a function of time. Red areas correspond to large hinges induced by oligomerization. Spontaneous aggregation and dissociation of TFAM oligomers as well as entrapment of oligomers/monomers due to sequence specificity are present in this example. (b) Monte Carlo simulation of 20 TFAMs interacting with the average PMF presented in Figure 2. Picture is similar to a, but for considerably larger length and time scales, thus allowing direct comparison with experiments^17^,^24^.

**Figure 4:**
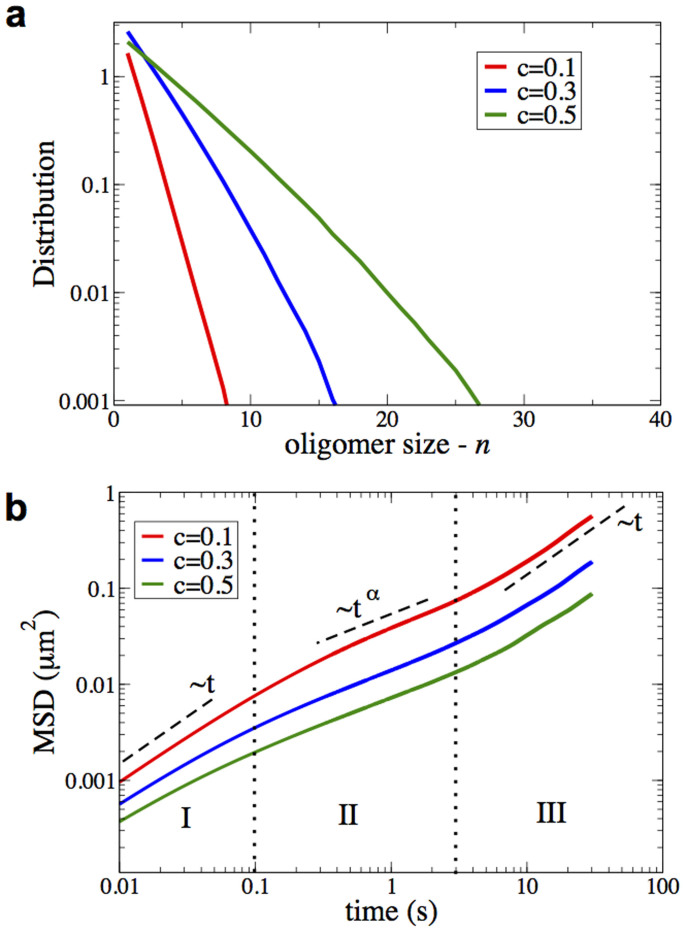
Statistics and dynamics of TFAM oligomerization. (a) Distribution of oligomer size for three different values of fractional coverage of DNA by TFAM, *c*. Red line corresponds to *c* = 0.1, blue to *c* = 0.3 and green to *c* = 0.5. The number of large flexible hinges in each oligomer is *n* − 1. (b) Log-Log plot of the mean square displacement (MSD) as a function of time for the same values of coverage as in a. The vertical lines define three areas of different diffusivity. The linear behavior at short (I) and long times (III) is due to protein oligomerization and crowding effects, respectively^37^. At intermediate times (II), transient arrested states (caging) result in sub-diffusive behavior (*α* < 1). Results for both a and b are obtained by MC simulations of protein dynamics in a 3000 bp long DNA.

To obtain a qualitative picture of the dynamics of the system, we perform a standard Langevin simulation of 10 TFAMs in an 1000 bp (~0.33 μm) long mtDNA sequence. Figure 3a shows that the position of all TFAMs as a function of time is qualitatively similar to that in the experimental work of Ref. 24. In agreement with these authors, protein oligomerization and dissociation events, oligomer/monomer diffusion, and entrapment due to sequence specificity are also present in our numerical simulations. However, even the Langevin dynamics of a 1D model cannot access scales similar to the experimental ones (~sec and ~10 μm). To overcome this limitation, we implement standard Monte Carlo (MC) simulations of multiple TFAMs interacting with the average PMF shown in Figure 2 (Methods). Figure 3b shows the MC time evolution of a similar system to that in Figure 3a, but for scales directly related to the experimental ones. We emphasize, however, that MC simulations do not take into account the sequence of the DNA; thus, entrapment due to sequence specificity is not observed. Figure 4a shows the distribution of the oligomer size, *n*, for different values of the coverage, *c*, of DNA by TFAM. The number of large flexible hinges is simply *n* − 1. In Figure 4b we show the mean square displacement (MSD) of the TFAMs as a function of time for the same values of *c*. We observe three distinct regions. The first region (I) describes the cluster diffusivity prior to collisions. It is purely linear and the slope determines the diffusivity of the system, which scales inversely with the oligomer size appropriately weighted by the distribution of sizes presented in Figure 4a. For intermediate times (region II) the system considerably slows down due to caging effects, i.e. clusters are arrested by nearby clusters and thus only the dynamics within the cage is described. The long time limit (region III) also shows linear behavior that is due to crowding effects^37^. A similar transition from the ballistic regime of individual proteins to region I is also observed but not shown in this plot. All three regimes affect DNA packaging. The slower a TFAM oligomer is, the more stable and long-lived are the developed large hinges.

## Discussion

In this work, we showed that protein-induced local melting of DNA is an alternative allosteric mechanism to drive protein assembly. Below, we discuss how TFAM may use such a mechanism to regulate DNA compaction and transcription initiation. Although we focus on TFAM, we believe that other proteins of the HMG family may also use the same allosteric signaling to control DNA functions [in preparation].

**Figure 5:**
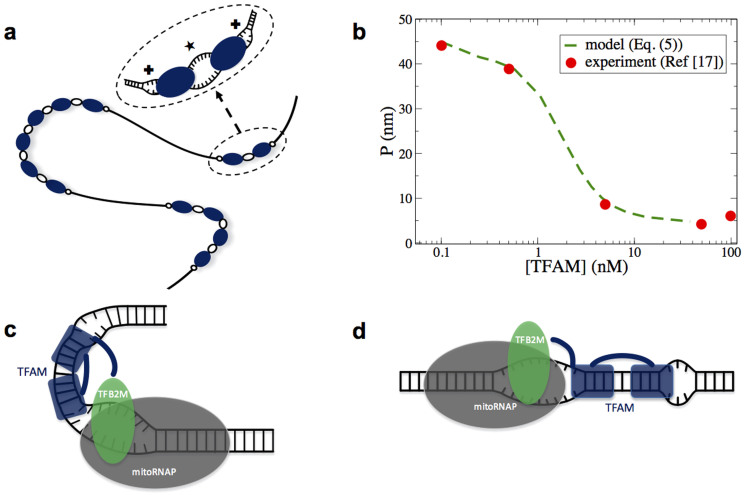
The Role of TFAM-induced bubbles on DNA compaction and transcription initiation. (a) Effect of large hinge formation (star) due to TFAM oligomerization on DNA compaction. Large oligomers bend DNA more effectively. Note that small hinges (crosses) develop only when the oligomers diffuse extremely slowly. For monomers, small hinges are evident only upon specific binding (see below). (b) Persistent length of the DNA as a function of TFAM concentration, calculated using equation (5), in comparison with experimental results of Ref. 17. (c) Schematic illustration of LSP activation by TFAM. TFAM first assists TFBM2 and mitoRNAP to excite the transcription bubble and then a nucleation of the bubble induced by Box A and the transcription bubble stabilizes the transcription machinery. The small bubble at the end of Box B and the large bubble at the end of Box A act as flexible hinges allowing the DNA to rotate. The interaction between the tail and TFB2M can stabilize a U-turn in agreement with the experimental works of Ref. 18. (d) Same as in c but for the HSP activation. Note that in this case the interaction of the tail with TFB2M prevents rotation around the large flexible hinge and thus a U-turn is not possible, as also observed in Ref. 18.

Our simulations support the hypothesis that the mechanism of flexible hinges induced by TFAM oligomerization underlies the compaction of mtDNA by TFAM (see Fig. 5a). More accurately, as we show in this work, the concept of spontaneously generated *diffusive flexible hinges* with *finite lifetimes* is closer to the experimental picture of Ref. 24. Although both small and large hinges contribute to DNA compaction, large hinges are expected to have a more significant impact, since they are energetically more favorable and diffuse much more slowly. Small hinges are more effective in specific binding or entrapment due to sequence specificity. Assuming that the compaction of DNA is primarily regulated by large hinges, one can use the distribution of hinges presented in Figure 4a to estimate the persistence length of mtDNA for different concentrations of TFAM. If *P*_0_ and *P*_p_ are the persistence lengths of the mtDNA in the absence of TFAM and fully covered by TFAMs, respectively, then, the persistence length for any value of coverage *c* can be estimated by^38^
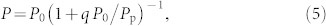
where q is the number of hinges for a given *c* normalized by the maximum number of hinges, *L*_DNA_/*σ*_eff_, in a DNA molecule. According to Ref. 17 *P*_0_ = 45 nm and *P*_p_ = 3.9 nm. The value of *q* can be calculated from the distribution of hinges presented in Figure 4a. Figure 5a compares the persistence length estimated by equation (5) with the experimental result of Ref. 17. TFAM coverage is converted to TFAM concentration through the McGhee-von Hippel formula^39^ using a cooperativity factor of *ω* = 70, equilibrium protein–DNA binding constant *K* = 1.6 × 10^6^ *M*^−1^ (see Ref. 17) and *σ*_eff_ = 31 bp as the protein footprint. Although one should consider the full 3D problem [work in progress], we see that a simple mathematical model can still provide estimates in good agreement with experimental observations.

Appropriate modifications of equations (2) and (3) can also describe the effect of TFAM mutants on compaction efficiency^18^. TFAM mutants missing either Box A or Box B exhibit significantly lower compaction efficiency. Based on our model, with such mutants, which can be described by eliminating one of the two terms of *V*_2_, only dimerization is possible. This leads to a significantly lower number of flexible hinges, which ultimately reduces the flexibility of the DNA. Mutants with a modified linker (L6) present similar behavior and reduce the efficiency of compaction by approximately the same amount. It appears, as we explain in more detail below, that the L6 mutant reduces only the ability of Box A to unwind the DNA molecule, which can be interpreted in our model by using an asymmetric strength in the expression of *V*_2_. This modification also provides only dimers, thus reducing the mutant's ability to compact the DNA in a way similar to that of a mutant that is missing Box A. Finally, the dimer mutants presented in Ref. 18 also show a significant reduction in compaction efficiency. Dimer mutants do not interact strongly with each other because their surface has been modified. Their ability, however, to locally unwind the DNA on both sides of the TFAM is preserved. In our model, dimer mutants can be interpreted by increasing the repulsive potential of equation (3). The resulting PMF will have a smaller depth, which will finally reduce the lifetime of TFAM oligomerization and, as a result, reduce the bendability of the DNA. However, it has to be mentioned that, even in the case of a perfect dimer mutant, which is the limit of hard spheres, caging effects can also provide some large flexible hinges that could contribute to DNA compaction^37^.

According to our hypothesis, in an intermediate step, TFAM-induced bubbles first assist TFB2M and mitoRNAP to excite the transcription bubble and then a coalescence of the two bubbles stabilizes the transcription machinery. The size of the resulting bubble (~10 bp) is consistent with the typical size of transcription bubbles^25^,^40^,^41^,^42^. Since TFAM can melt DNA in both Boxes A and B, the same mechanism can be used to activate transcription at LSP and HSP1, as presented in Figures 5c and 5d, respectively. This leads to the creation of a large hinge on the promoter's side and a small hinge on the other side of TFAM. These two hinges in combination with the strong interaction between the TFAM tail and TFB2M can also explain why a U-turn is present in LSP and not necessarily present in HSP1 (see Figs. 5c and 5d)^18^. It is worth noting that dimer mutants do not affect transcriptional activity, which is in accordance with our hypothesis, mentioned above, that dimer mutants preserve the ability to locally melt the double strand. Additionally, it indicates that a dimer mutant modifies the repulsive interaction between TFAMs but not necessarily the repulsion between a TFAM and other proteins. In Ref. 18, the L6 mutant appears unable to activate transcription in LSP. According to our hypothesis, this observation implies that L6 does not melt the DNA in Box A and consequently cannot recruit TFB2M and mitoRNAP by using the mechanism described above. That L6 can unwind mtDNA only at Box B is further supported by the fact that L6 activates HSP1. It can also activate LSP only upon interchanging the box domains of TFAM^18^.

## Methods

### Langevin Dynamics

The Langevin equations of motion for the base pairs and proteins are, respectively,
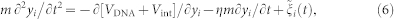
and
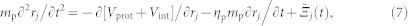
where *i* = 1,2, … *N*_b_ and *j* = 1,2, … *N*_p_. Here, *N*_b_ represents the total number of base pairs and *N*_p_ the number of proteins sliding on the DNA. The molecular weight of a base pair is *m* = 600 Da and for a TFAM is *m*_p_ = 29 kDa. The potential energy *V*_DNA_, *V*_int_, and *V*_prot_ are given by equations (1)–(4). The parameters of *V*_int_ and *V*_prot_ were fitted to reproduce the experimentally observed cooperativity factor of TFAM binding affinity. Specifically *A*_1_ = 0.025 eV, *A*_2_ = 0.13 eV, *γ*_1_ = 2 Å^−1^, *γ*_2_ = 0.225 Å^−2^, and *ε* = 0.125 eV. The phenomenological Langevin friction coefficients are *η* = 0.1 ps^−1^ (for the base pairs), and *η*_p_ = 0.1 ps^−1^ (for the proteins). The stochastic forces 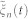 and 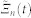 are modeled as Gaussian random noise with covariances of 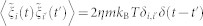 and 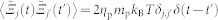, respectively, where *T* is the temperature and *k*_B_ is the Boltzmann constant. The equations of motion were integrated numerically using a second order Runge-Kutta method^43^ with periodic boundary conditions. The time step *dt* = 0.001 ps ensured stable and accurate simulations. For each simulation the system was initially thermalized for 50 ns before starting to monitor the trajectories.

### Potential of Mean Force

The effective force between two proteins was probed through a harmonic spring of strength *k* connecting the centers of the two proteins. The equilibrium length *L*_0_ of the spring varied between *σ* − 3 bp and *σ* + 100 bp. For each *L*_0_, we performed 100 independent Langevin simulations of 1 μs duration to compute the average inter-protein distance 〈*r_ij_*〉. The mean force between the two proteins was estimated by *F* = *k*(*L*_0_ − 〈*r_ij_*〉). The potential of mean force (PMF) was then calculated by a simple integration of the computed force in space.

### Monte Carlo Simulations

TFAM proteins interact through the average PMF presented in Figure 2 (green line). In each MC step all proteins are moved by 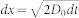, where *D*_0_ = 0.08 *μm*^2^/*s* is the reference diffusivity as calculated in Ref. 24 and *dt* = 0.003 *s* is the time step of an MC step. Each trial move of a protein is accepted/rejected based on the standard Metropolis algorithm. Before each MC sampling the system was thermalized for 1 sec. All results were obtained by averaging 100 independent MC simulations.

## Author Contributions

N.K.V. conceived and designed the research, carried out the simulations, and analyzed the results. J.J.T. carried out part of PMF calculations. V.S.M., A.R.B. and K.O.R. contributed with the interpretation of the data and gave conceptual advice. All authors wrote the manuscript.
